# Alveolar Proteinosis in COVID-19: Clinical Case

**DOI:** 10.1155/2022/1842566

**Published:** 2022-10-22

**Authors:** Silvia Martin Bote, Maria Angeles Herrera Morueco, Beatriz Arias Arcos, Javier García Lopez, Maria Belen Lopez-Muñiz Ballesteros

**Affiliations:** ^1^Department of Respiratory Medicine, Infanta Leonor University Hospital, Madrid, Spain; ^2^Department of Internal Medicine, Infanta Leonor University Hospital, Madrid, Spain; ^3^Department of Respiratory Medicine, Gregorio Marañón General University Hospital, Madrid, Spain

## Abstract

Pulmonary alveolar proteinosis (PAP) is a rare, diffuse lung disease characterized by accumulation of lipoprotein in lung surfactant in the alveolar space and terminal bronchioles, leading to impaired gas exchange and arterial hypoxemia. We present the case of a 51-year-old woman who was admitted with a diagnosis of severe SARS-CoV-2 pneumonia. Her condition did not improve with corticosteroids. A chest CT scan revealed ground-glass opacities in all lung lobes, with septal thickening. A differential diagnosis was proposed with other diseases. Bronchoscopy revealed milky bronchoalveolar lavage fluid, and staining with periodic acid–Schiff was positive, thus indicating PAP. Therefore, the patient underwent whole lung lavage, which led to clinical, radiological, and functional improvement. In the context of the COVID-19 pandemic, differential diagnosis ensures that appropriate attention is given to less prevalent entities such as PAP.

## 1. Introduction

Clinical characteristics and radiological pattern of SARS-CoV-2 infection have been widely described in the literature [1]. However, findings are not pathognomonic and are observed in other diseases such as viral pneumonia (influenza A and B, adenovirus, and cytomegalovirus), interstitial pneumonia (organizing pneumonia, chronic eosinophilic pneumonia, and hypersensitivity pneumonia), *Pneumocystis jirovecii* pneumonia, diffuse alveolar hemorrhage, pulmonary edema, and pulmonary alveolar proteinosis (PAP) [2], an infrequent, underdiagnosed disease. The most common type of PAP is idiopathic; although PAP can also occur as a secondary phenomenon, resulting from inhalation of substances and infectious agents, and it has been associated with hematological malignancies [3].

We present the case of a patient admitted for pneumonia due to SARS-CoV-2 infection who developed PAP, and we describe the approach undertook to determine whether PAP was a consequence of COVID-19 or a fortuitous event.

## 2. Case Presentation

The patient is a 51-year-old woman from Peru who had been living in Spain for the previous 2 years. Her medical history was unremarkable, and she had no toxic habits. She worked as a seamstress and denied exposure to organic or inorganic toxic pulmonary agents.

In the last year, she reported dyspnea on moderate effort and coughed white mucus, although she did not consult a physician.

**Figure 1:**
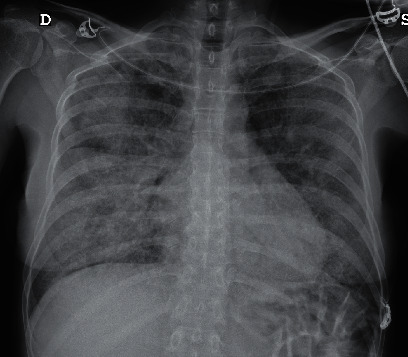
Chest X-ray on admission. Note the bilateral interstitial pattern with bilateral diffuse lung involvement.

**Figure 2:**
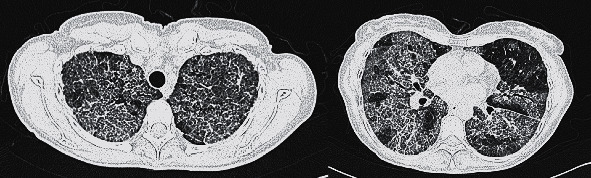
CT scan of the patient during admission showing extensive pulmonary involvement with a crazy-paving pattern (septal thickening and ground-glass opacity) in a patchy distribution throughout the lobes.

She went to the emergency room with severe dyspnea and asthenia. The patient's vital signs were tachycardia, tachypnea, peripheral oxygen saturation was 65% on room air, and afebrile. The polymerase chain reaction (PCR) test was positive for SARS-CoV-2. A chest X-ray revealed predominantly right-sided bilateral alveolar interstitial infiltrates (Figure 1). Laboratory investigations showed lymphopenia (1100 cells/*μ*L), high D-dimer (590 *μ*g/L), lactic dehydrogenase of 849 units/L, and C-reactive protein of 73 mg/L. The patient was transferred to the intensive care unit, where she was treated with high-flow oxygen nasal cannula and dexamethasone 20 mg for 5 days. Three days later, she was transferred to the conventional ward. At this point, the patient had tachypnea, required oxygen at 5 lpm with nasal cannula, with marked desaturation on minimal effort and persistence of the radiological interstitial infiltrate. Computerized tomography (CT) angiography of the lung revealed extensive bilateral involvement consisting of a crazy-paving pattern with septal thickening, ground-glass opacities, and diffuse lung involvement (Figure 2). Bronchoscopy yielded milky bronchoalveolar lavage (BAL) fluid. Histopathology smears revealed abundant proteinaceous material and macrophages. The periodic acid–Schiff (PAS) reaction was positive, indicating alveolar proteinosis. The microbiological culture was remarkable only for the positive SARS-CoV-2 result (PCR).

A systemic study enabled us to rule out underlying diseases such as immunodeficiency (HIV), autoimmune disease, and blood dyspraxia. Pulmonary function testing showed a mild restrictive ventilatory defect with small lung volumes: forced expiratory volume in 1 second (FEV_1_), 1530 L (71%); forced vital capacity (FVC), 1570 L (62%); FEV_1_/FVC, 97; total lung capacity, 3230 mL (76%); inspiratory capacity, 1300 mL (66%); residual volume, 490 mL (31%); and reduced diffusing capacity of the lung for carbon monoxide (DLCO), 28%.

**Figure 3:**
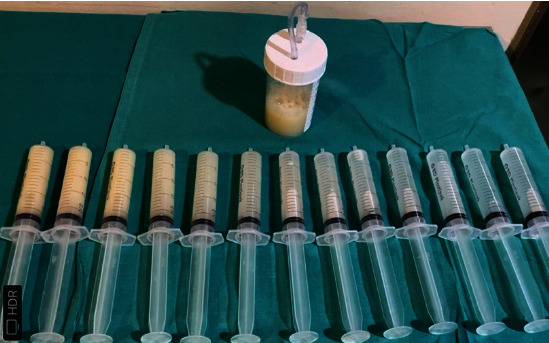
Samples of the saline solution obtained during WLL. Note how the colour clears during the procedure.

A whole lung lavage (WLL) was programmed. Given the severity of lung involvement, a sequential lavage of both lungs was performed in the same session in combination with extracorporeal membrane oxygenation (ECMO). No complications were reported [4] (Figure 3).

**Figure 4:**
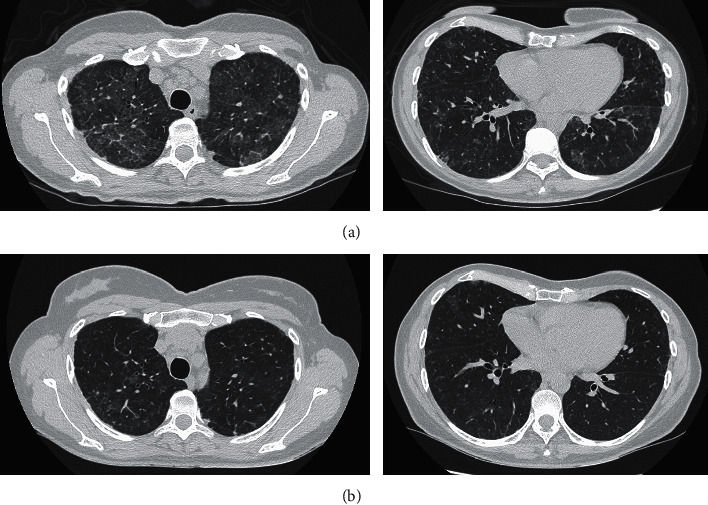
(a) One month after WLL. (b) Five months after WLL. Significant decrease in ground-glass opacity and septal thickening, although discrete patchy opacities persist in the lower fields. Note the interlobular thickening of diffuse distribution.

One month after the WLL took place, the spirometry revealed an improvement of 14% in DLCO and a significant improvement in the crazy-paving pattern on the CT scan, although all the lung lobes continued to be involved (Figure 4). A serology with anti-granulocyte-macrophage colony stimulating factor (anti-GM-CSF) antibodies was requested, yielding a doubtful cut-off of 2.4 U/mL (positive, >5). Testing for mutations in the *CSF2RA* and *CSF2RB* genes was negative.

As pulmonary lesions continue to display on the CT scan, a cryobiopsy was performed to rule out other diseases associated with well-structured lung parenchyma. The histopathology revealed no abnormalities that would confirm the diagnosis.

Five months after the initial diagnosis, a new CT scan revealed a considerable improvement in lung parenchyma (Figure 4) and normalization of pulmonary function, as follows: FEV_1_, 2040 L (98%); FVC, 2370 L (88%); FEV_1_/FVC, 86; and DLCO, 80%.

## 3. Discussion

PAP is a rare lung disease characterized by abnormal accumulation of PAS-positive lipoprotein in alveolar spaces and terminal bronchioles. The estimated incidence is 0.24-0.49 cases per million inhabitants, and the prevalence is 2.04-6.2 cases per million inhabitants [5]. Males are more commonly affected than females at a 2 : 1 ratio. The median age of diagnosis is 50 years [6]. It usually presents as progressive dyspnea and cough that may be accompanied by fever, pain, and/or hemoptysis, although a third of patients may be asymptomatic. PAP may be idiopathic, secondary, and congenital. Idiopathic PAP is the most common; it accounts for 90% of cases and generally has an autoimmune basis. Anti-GM-CSF antibodies cause macrophage dysfunction that result in impaired clearance of surfactant, leading to accumulation. This group also includes hereditary PAP (<1%) due to mutations in the GM-CSF receptor genes (*CSF2RA* and *CSF2RB*). Secondary PAP (5-10%) is associated with hematological cancers (lymphoma, leukemia, and myelodysplastic syndrome), immunodeficiency, inhalation of toxic substances, and infection. Congenital PAP is caused by mutations in genes involved in surfactant production and is the least common [3].

Abnormalities in macrophages and alveolar neutrophils increase the risk of opportunistic infections, which may affect onset of PAP and alter its course. Opportunistic infection is reported in 5% and 20% of cases, the most common agents being *Nocardia, Mycobacterium tuberculosis, Mycobacterium avium-intracellulare, Pneumocystis jirovecii,* Epstein-Barr virus, and cytomegalovirus [7, 8]. PAP has also been associated with influenza virus, although few cases have been reported [9]. There are very few reported cases of association between PAP and SARS-CoV-2 to date [10, 11]. Surbhi et al. described a patient with a diagnosis of autoimmune PAP who had received treatment with WLL and GM-CSF. The patient presented an infection with COVID-19, requiring admission to the ICU with an uncertain prognosis. Although a clinical worsening of the patient was described, there was no impact in the evolution of PAP.

In the case we have described, we cannot assign the patient to any specific group. Anti-GM-CSF antibody levels were unclear, probably because the test was performed 1 month after WLL. A decrease in levels after the technique has been reported [12].

Despite the uncertain result, we highly suspect primary PAP is exacerbated by SARS-CoV-2 infection, since the patient had mild symptoms (dyspnea and cough) prior to the infection. In addition, we have not found other causes to justify hereditary PAP (negative genetic study) or secondary PAP (no exposure to inhaled substances or known hematological diseases).

The most common pattern in CT scans in alveolar proteinosis is ground-glass areas superimposed on the thickening of the interlobular and intralobular septal lines, resulting in a crazy-paving pattern [13]. The most frequent radiological patterns for COVID-19 have been bilateral ground-glass opacities with or without consolidation and/or a crazy-paving pattern [2]. This can generate confusion and diagnostic delay, as in the case we have reported.

The diagnosis of PAP is usually based on a compatible medical history accompanied by typical CT images with milky bronchoalveolar lavage fluid and a positive PAS reaction [13]. Lung biopsy should be considered in patients whose radiological or bronchoalveolar lavage findings are not characteristic. This approach shows the alveoli to be occupied by acellular, amorphous, eosinophilic material (seen clearly with PAS staining), and foamy alveolar macrophages [6].

Mild and moderate forms of PAP require monitoring, since spontaneous resolution has been described. Severe PAP, on the other hand, is generally treated with WLL, which was first reported more than 40 years ago. This technique consists of selective intubation with a double lumen tube, instillation, and reabsorption of saline solution to remove accumulated surfactant [13]. In recent years, variations have been described with simultaneous use of ECMO in severe cases, enabling the sequential lavage of both lungs in the same session [4]. In the case above, the patient opted for this technique due to her poor DLCO (28%).

The therapeutic benefit of inhaled or subcutaneous GM-CSF analogs remains understudy. A favorable response has been reported in 50-60% of patients in clinical trials [4]. Data from a clinical trial show that rituximab did not improve reduced anti-GM-CSF antibody levels [14]. Lung transplantation is reserved for patients who have not responded to previous treatments. Experience is scarce, and recurrences have been observed in lung recipients [15].

PAP has a variable clinical course ranging from spontaneous resolution to death by pneumonia or respiratory failure [16]. The survival rate exceeds 80% at 5 years [17].

The patient we have described is currently being followed up as an outpatient. She remains asymptomatic and does not require oxygen therapy. Her spirometry results are normal, and CT shows almost complete resolution.

In conclusion, it is important to make a correct differential diagnosis of PAP with other conditions that may present clinical and/or radiological pictures similar to those of SARS-CoV-2 infection.

## Data Availability

The data used to support the findings of this study are included within the article.
